# Multidimensional Single-Cell Analysis of BCR Signaling Reveals Proximal Activation Defect As a Hallmark of Chronic Lymphocytic Leukemia B Cells

**DOI:** 10.1371/journal.pone.0079987

**Published:** 2014-01-29

**Authors:** M. Lia Palomba, Kelly Piersanti, Carly G. K. Ziegler, Hugo Decker, Jesse W. Cotari, Kurt Bantilan, Ivelise Rijo, Jeff R. Gardner, Mark Heaney, Debra Bemis, Robert Balderas, Sami N. Malek, Erlene Seymour, Andrew D. Zelenetz, Marcel R. M. van den Brink, Grégoire Altan-Bonnet

**Affiliations:** 1 Division of Hematology, Memorial Sloan-Kettering Cancer Center, New York, New York, United States of America; 2 Center Cancer Systems Biology, Memorial Sloan-Kettering Cancer Center, New York, New York, United States of America; 3 Program in Computational Biology, Memorial Sloan-Kettering Cancer Center, New York, New York, United States of America; 4 BD Biosciences, San Diego, California, United States of America; 5 Division of Hematology/Oncology, University of Michigan Health System, Ann Harbor, Michigan, United States of America; Institut National de la Santé et de la Recherche Médicale, France

## Abstract

**Purpose:**

Chronic Lymphocytic Leukemia (CLL) is defined by a perturbed B-cell receptor-mediated signaling machinery. We aimed to model differential signaling behavior between B cells from CLL and healthy individuals to pinpoint modes of dysregulation.

**Experimental Design:**

We developed an experimental methodology combining immunophenotyping, multiplexed phosphospecific flow cytometry, and multifactorial statistical modeling. Utilizing patterns of signaling network covariance, we modeled BCR signaling in 67 CLL patients using Partial Least Squares Regression (PLSR). Results from multidimensional modeling were validated using an independent test cohort of 38 patients.

**Results:**

We identified a dynamic and variable imbalance between proximal (pSYK, pBTK) and distal (pPLCγ2, pBLNK, ppERK) phosphoresponses. PLSR identified the relationship between upstream tyrosine kinase SYK and its target, PLCγ2, as maximally predictive and sufficient to distinguish CLL from healthy samples, pointing to this juncture in the signaling pathway as a hallmark of CLL B cells. Specific BCR pathway signaling signatures that correlate with the disease and its degree of aggressiveness were identified. Heterogeneity in the PLSR response variable within the B cell population is both a characteristic mark of healthy samples and predictive of disease aggressiveness.

**Conclusion:**

Single-cell multidimensional analysis of BCR signaling permitted focused analysis of the variability and heterogeneity of signaling behavior from patient-to-patient, and from cell-to-cell. Disruption of the pSYK/pPLCγ2 relationship is uncovered as a robust hallmark of CLL B cell signaling behavior. Together, these observations implicate novel elements of the BCR signal transduction as potential therapeutic targets.

## Introduction

Chronic lymphocytic leukemia (CLL) results from the accumulation of mature monoclonal CD5^+^ B cells in the bone marrow, lymphoid organs and peripheral blood. CLL B cells are characterized by low expression of surface CD20 and co-expression of CD19 and CD5 [1]. While some patients have rapidly progressive disease that is characterized by early need for treatment, resistance to chemotherapy and short survival, others have a stable, indolent course over many years and often succumb from other causes [2]. In patients with evidence of clinically indolent course, treatment is generally delayed and a period of “watch and wait” is typically indicated [3]. Over the past decade molecular and cellular prognostic markers have been identified that correlate with response to treatment and/or overall survival (among which one of the most accurately predictive is the immunoglobulin heavy chain (IGHV) gene mutation status), though the discriminatory power of these known prognosticators is not absolute [4]–[8].

At the cellular level, clonal expansion of B cells depends on the efficient propagation of signal from the cell membrane to target genes following antigenic stimulation of the BCR [9], [10]. It has been proposed that unmutated surface immunoglobulins in CLL are more responsive to antigenic stimulation, resulting in strong BCR-mediated signal transduction and induction of anti-apoptotic proteins such as XIAP and MCL-1 [11]–[13], while CLL cells with mutated IGHV more closely resembles anergic B cells [14], with incomplete responsiveness through the BCR pathway and induction of tolerogenic signals. CLL B cells have been described to have constitutive activation of several members of the BCR signalosome. For instance, levels of phosphorylated Lyn and Syk have been shown to be higher in CLL cells [15], [16]. Similarly, the PI3K/Akt pathway has been shown to be aberrantly activated in CLL cells [17], [18]. BCR signaling aberrancy has been shown to correlate with prognostic clinical parameters or disease stage at the time of diagnosis. Recent work from the Jumaa laboratory identified a possibly parallel BCR activation mechanism, whereby a structural motif of the CLL BCR drives antigen-independent autonomous signaling [19]. Regardless of the initial activating event, the BCR pathway is clearly an ideal target for new drug development in CLL. Small molecule inhibitors of the BCR signaling pathway are demonstrating remarkable activity in clinical trials. The target specificity, off-target activity and exact mechanism of action of these novel drugs, however, are not completely understood at this time [20]–[25].

Single-cell network profiling is a method that allows the investigation of cell signaling events with single-cell resolution and requires minimal sample manipulation [26]. Using combined immunophenotyping and multiplexed phosphospecific flow cytometry Irish *et al.* identified a subpopulation of lymphoma cells with impaired BCR signaling from tumor samples of patients with follicular lymphoma [27], [28]. A negative impact of these low-responding cells on patients overall survival was noted. More recently, using a similar methodology, Palazzo *et al.* demonstrated that CLL B cells could be stratified into two groups depending on the efficiency of BCR signal amplification caused by hydrogen peroxide (H_2_O_2_), a broad tyrosine phosphatase inhibitor. Furthermore, in vitro response to the nucleotide analog F-Ara-A by primary CLL cells was highly associated with the ability of CLL B cells to undergo peroxide-augmented signaling [29].

We applied phosphospecific flow cytometry to study the heterogeneous response of stimulated B cells with the goal of identifying BCR pathway signaling signatures that correlate with the disease and with its degree of aggressiveness. We hypothesized that this method would allow the identification of CLL-specific signaling behavior that would be highly predictive of the presence of disease in a B cell population. To do this, we stimulated the cells with anti-IgM and H_2_O_2_, a highly standardized, widely published method for probing the signaling pathway of B cells [28]–[30]. We found that the development of a sophisticated computational method was essential to analyze the phospho-flow data in its multidimensional nature, rather than as a series of individual results.

## Materials and Methods

### Sample preparation

We used samples from 67 CLL patients and 10 healthy volunteers who signed informed consent to have their sample used for research purposes, in accordance with the Declaration of Helsinki and approval by the Memorial Sloan-Kettering Cancer Center (MSKCC) institutional review board. Treated and untreated CLL patient samples were randomly selected. Patients were not treated uniformly under a specific clinical trial. Clinical characteristics are described in **Supplementary Table S1**. 38 additional samples from another institution (a generous gift of Dr. Sami Malek, University of Michigan) were used as a validating cohort for BCR signaling analysis, or combined with the MSKCC samples for selected analysis.

### Isolation, storage, and thawing of primary cells

Peripheral blood mononuclear cells (PBMCs) were isolated using density gradient separation (Ficoll-Paque Plus; GE Healthcare) and frozen within 6 hours from collection, without further manipulation. For signaling analysis, cells were thawed and washed in RMPI+10% FBS. Mononuclear cells were resuspended at 10^7^ cells/mL, and allowed to rest at 37°C for up to 2 hours.

### Immunophenotyping and total cytoplasmic Zap-70 analysis

For immunophenotyping, cells were stained anti-CD3, -CD5, -CD19, -CD20 (cytoplasmic), and -CD38 (all BD-Biosciences), and anti-IgM F(ab′)_2_ (Biosource International). For cytoplasmic Zap-70 analysis, resting cells were fixed with a 1.6% paraformaldehyde solution for 10 minutes at 37°C then permeabilized with cold methanol at −20°C for 10 minutes. Cells were then washed with PBS with 2% FBS and stained with anti-Zap-70 antibody (clone 1E7.2, BD Biosciences) for 30 minutes. Cells were washed once with PBS+2% FBS, and collected on an LSRII cytometer (BD Biosciences).

### IGHV gene sequencing

DNA was amplified using BIOMED-2 multiplex PCR assays with consensus primers that have been previously developed and standardized for detecting clonally rearranged immunoglobulins [1]. The DNA was sequenced using Applied Biosystem's BigDye Terminator v3.1 Cycle Sequencing Kit. Mutation levels were analyzed by comparison to known germline sequences using VBASE.

### BCR stimulation

After thawing, cells were rested in FBS-containing medium, then stimulated by adding goat polyclonal anti-IgM F(ab′)_2_ (Biosource International) to a final concentration of 10 µg/mL at 37°C. A resting time of 2 hours was chosen based on time course experiments that demonstrated that, while a 15 minute resting time was sufficient to achieve maximum stimulation in highly responding samples, continued incubation in FBS-containing medium up to 2 hours provided enhanced phosphoresponses in the lower responders (**Supp. Fig. S1A**). Addition of hydrogen peroxide (H_2_O_2_) further enhanced observed phosphoresponses in non-maximally activated samples, in a dose-dependent manner (**Supp. Fig. S1B and S1C**). A working concentration of 3.3 mM H_2_O_2_ was chosen. Fixation was achieved with the addition of pre-warmed paraformaldehyde (BD Biosciences) to a final concentration of 1.6%. Cells were fixed for 10 minutes at 37°C, permeabilized with cold methanol at −20°C for 10 minutes, and stored at −80°C until staining step, no longer than 48 hours.

### Phosphospecific flow cytometry

Fixed and permeabilized cells were washed once with PBS with 2% FBS. An antibody mix containing conjugated phosphospecific antibodies was added, and incubated for 30 minutes at room temperature. Phosphospecific Alexa Fluor 488 and Alexa Fluor 647, or R-PE-conjugated antibodies (all from BD-Biosciences) against pBLNK(Y84), pBTK(Y551)/Itk(Y511), pERK1/2(T202/Y204), pPLCγ2(Y759) and pSYK(Y348) were used. Detection of PBMC subsets was achieved with Pacific Blue-conjugated anti-CD3 (clone UCHT1, BD Bioscience), PerCPCy5.5-conjugated anti-CD20 (clone H1, BD Biosciences), and PE-Cy7-conjugated anti-CD5 (clone L17F12, BD Biosciences) antibodies.

### Data analysis

Flow cytometry data were acquired on an LSR II Flow cytometer (Beckton Dickinson). After gating out doublets and dead cells, the CD3^−^/CD20^+^ gate was used to identify B cells within the healthy PBMCs. The clonal CD20^low^/CD5^+^/CD3^−^ population of each patient with CLL was manually gated.

Phosphoresponses for kinase X (pX = pBLNK, pBTK, ppERK, PLCγ2, or pSYK) were quantified by computing the frequency **F**(X) of cells whose Mean Fluorescence Intensity (MFI) for stimulated cells was 99% higher than the MFI for unstimulated cells. To reduce the dimensionality of the data, we applied a partial-least square regression (PLSR) using the Matlab statistical toolbox (Mathworks): the **F**(X) matrix for all phosphoresponses of all the samples (CLL or healthy controls) were pooled together in one matrix and linearly-regressed against a one-dimensional matrix of 0 for CLL patients and 1 for healthy individuals. The linear combination of **F**(X) yielded a one-dimensional variable V_PLSR_.

## Results

### CLL B cells exhibit wide variability in their responsiveness to BCR stimulation and pathological dysregulation of proximal signaling components

PBMCs from CLL patients and healthy volunteers were isolated by density gradient without further B-cell separation. All samples were collected, frozen and thawed following a uniform protocol to prevent inter-sample variations due to handling. BCR-mediated activation with anti-IgM and H_2_O_2_ was optimized to a stimulation time of 4 minutes.

In the CLL samples, CD20^+^/CD5^+^ cells represented a variable proportion of the total cells for each individual patient, depending on the extent of the circulating CLL clone. As expected, the mean fluorescence intensity (MFI) of the CD20 staining was generally dim, though some samples were strongly CD20^+^. Similarly, CD5 expression varied among patients.

**Figure 1:**
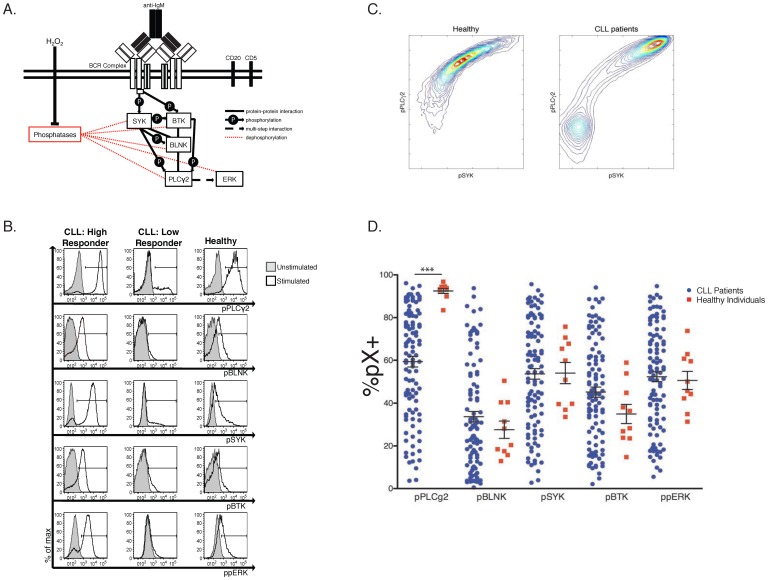
Phosphoprofiling of the Proximal BCR Signaling Pathway Uncovers High Variability in BCR Signaling Pathway Behavior in CLL B cells. A. Simplified diagram of the BCR signaling pathway components investigated in this study. B. Representative histograms of phosphoresponses of B- cells for two CLL patients (a high and a low responder) and one healthy individual. CLL and healthy B cells were gated as described in the methods section. Stimulated samples (anti-IgM+H_2_O_2_) are indicated by a black line, unstimulated samples are shaded grey. C. Contour maps of the average B cell population: For each cohort, CLL and healthy, the cellular phosphoresponse fluorescence intensity values are averaged and viewed two dimensionally. Bimodality in the phosphoresponse of CLL B cells can be seen for the pairwise pPLCγ2 vs. pSYK) (see also Supp. Fig. S2 for other combinations of phosphoresponses). Healthy B cells show modest variability within the B-cell population, while the CLL B cells can be distinguished by their all-or-none response. D. Following IgM crosslinking, phosphoresponses are shown as the percentage of responding cells for each patient over an unstimulated matched control. This view of the wide, continuous range of each CLL patients' phosphoprofile highlights the high variability in the behavior of the BCR signaling pathway. Only the pPLCγ2 response showed statistical significance when comparing healthy to CLL cells (***p<0.0001).

At baseline, both CLL and healthy B cells had no or minimal constitutive activation of each of the upstream or downstream BCR signaling proteins analyzed. Addition of H_2_O_2_ amplified the stimulatory effect of BCR cross-linking, significantly more so in CLL B cells than in healthy controls (**Supp. Fig. S1C**). This confirms that CLL B cells are overall anergic, possibly because of an excess of phosphatase activity that can be blocked upon H_2_O_2_ treatment [31]–[33]. Following H_2_O_2_ exposure and BCR cross-linking for 4 minutes, we analyzed the intensity of activation, measured as MFI of five specific phosphoepitopes of a selected number of BCR signaling pathway members (**Fig. 1A**). The detectable fraction of cells responding with phosphorylation of SYK, BLNK, BTK, PLCγ2, or ERK1/2 was highly variable among the 105 CLL patient samples analyzed. Examples of “high responder” and “low responder” CLL samples, compared with B cells from one healthy individual, are shown in **Fig. 1B**. Preliminary analysis of sample phosphoresponses revealed strong correlation between each pairwise pX and pY (i.e. pPLCγ2 and pSYK) (**Supp. Fig. S2A, S2B**). Contour plots of MFI illustrate bimodal patterns of stimulation within all CLL patient samples; bimodality is absent from healthy B cell populations (**Fig. 1C** **, Supp. Fig. S2C**). Therefore, within a single population, the B cells of healthy individuals show modest variability (yielding mostly uniformly intermediate responses), while the CLL B cells can be distinguished by their all-or-none response (visualized by two populations separated by phosphoresponse). Importantly, we checked that cell viability was homogeneous among all samples, thus variable bimodality of the B cell response to stimulation is not an artifact of our cell preparation protocol (**Supp. Fig. S3**). Bimodality of the stimulated response is a novel observation of BCR signaling pathway dynamics in CLL patients [34].

Comparison of the percentage of responding cells over the corresponding unstimulated control in all CLL samples and healthy controls is shown in **Fig. 1D**. This representation reveals the wide, continuous range of CLL patients' phosphoprofiles and highlights the high variability in the responsiveness of the BCR signaling pathway in this patients' population. Importantly, this analysis reveals that while CLL cell populations all have some degree of bimodality in their signaling response, the fraction of the population with a stimulated phenotype is widely variable between CLL patients. Surface IgM staining revealed no correlation between expression of IgM and magnitude of signaling response, indicating that the changes in %pX^+^ among CLL patients is due to alterations in the signal transduction mechanism (data not shown).

Using this simple comparison of each isolated phosphoresponse, only the PLCγ2 activation showed statistical significance when comparing healthy to CLL samples (p<0.0001) (**Fig. 1D**). Analysis of the percentage of pX^+^ and of the MFI values of all phosphoresponses in every possible pairwise combination shows good linear correlation of the entity of phosphoresponses, and again highlights the greater variability observed in CLL patients (**Supp. Fig. S2A, S2B**).

### Multidimensional phosphoresponse signatures partition CLL patients into clinically relevant subgroups

**Figure 2:**
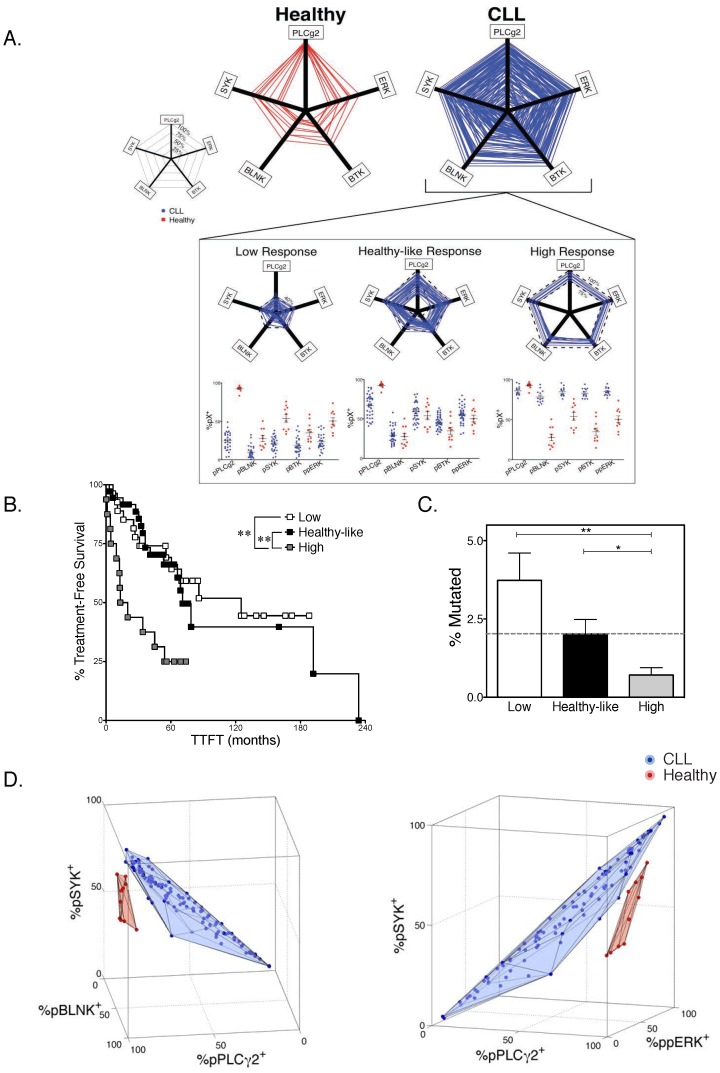
Variability in the Signaling Profile Allows Partioning of CLL Patients Into Distinct Prognostic Groups. A Graphic representation of all five phosphoresponses for CLL patients (blue, n = 110) as compared to the healthy controls (red, n = 11). A pentagon was created for each sample by connecting the percent of responding cells (over unstimulated control) recorded on the axes. A wide variability in extent of response was observed in the CLL group, encompassing all possible level of responsiveness. Three distinct groups of CLL could be defined based on the responsiveness (inset): patients with uniformly low response (left, at least 4 of the 5 pX^+^<40%), patients with uniformly high response (right, at least 4 pX^+^>75%), and patients with intermediate, healthy-like response (middle, at least 4 pX^+^ within the range of healthy samples). This groups of patients yielded n = 28 among low-responders, n = 36 among intermediate (healthy-like) responders and n = 17 among high-responders. B Kaplan-Meyer curves showing time from diagnosis to first treatment (TTFT) was plotted for the three distinct CLL subgroups described in D. Patients whose phosphoprofile consists of uniformly high phosphoresponses had a statistically significant (p<0.01) shorter time to first treatment (TTFT). Furthermore, high responders had a larger fraction that had required treatment (p<0.04). Those patients whose %pX^+^ values were uniformly low or similar to healthy individuals across the 5 responses studied had a significantly longer time to first treatment, and fewer patients within this cohort have required treatment at the time of this analysis. C Distinct IGHV status (% deviation from germline sequence) is also observed between these three subgroups. The dashed line indicates the 2% cutoff for mutational status, mutated vs. unmutated. Mean ± Std. Error % Mutated: Healthy-like = 2.02±0.43; High = 0.73±.21; Low = 3.9±.86. D Three-dimensional visualization of three phosphoresponses ([%pPLCγ2^+^, %pBLNK^+^ and %pSYK^+^], or ([%pPLCγ2^+^, %pSYK^+^ and %ppERK^+^]) for (blue) all CLL and (red) healthy samples. Each panel demonstrates the clear separation of the healthy patient samples from the CLL patient samples solely by virtue of the combined %pX^+^ values for the three phosphoresponses.

For more than 75% of the CLL cases analyzed, CLL B cells were either *uniformly* high-responders to IgM cross-linking (n = 17/105), low-responders (n = 28/105), or healthy-like with intermediate response (n = 36/105). Hence, we defined a five-dimensional filter based on the amplitude of the signaling responses (**Fig. 2A**) for these three populations and tested their ability to match known indicators of clinical outcome (time to first treatment, TTFT, or mutational status of IGHV genes).

Patients whose CLL B cells responded to IgM cross-linking with high BCR responsiveness required treatment after shorter periods of expectant monitoring (50% of cases treated by 12.4 months), compared to patients whose CLL B cells exhibited intermediate (healthy-like)-responsiveness (26.9 months) or low-responsiveness (36.3 months) (p<0.01). Hence, it appears that stronger B cell signaling response to BCR cross-linking in CLL B cells when compared to healthy B cells correlates with the severity of the disease (**Fig. 2B**). We also found that the degree of BCR responsiveness inversely correlated with the percentage of mutation in the IGHV genes compared to the germline BCR (**Fig. 2C**). Specifically, patients with high BCR responsiveness were more likely to have unmutated IGHV genes (using the standard 2% mutation cut-off compared to germ-line sequences), while patients with low responsiveness had overall mostly mutated IGHV genes. IGHV genes mutation rate in patients with intermediate, healthy-like responsiveness fell, on average, on the 2% cutoff. When comparing clinical parameters of disease aggressiveness (TTFT, treatment status, ZAP70 abundance) to individual phosphoresponses only pPLCγ2 correlated significantly with treatment status (**Supp. Fig. S4**), thus further supporting the multidimensional view of the BCR signaling pathway as an independent indicator of disease state. Indeed, visualization of the phosphoresponses in higher dimensions is a powerful tool to resolve disease status between a CLL and healthy B cell. Three-dimensional visualization of three phosphoresponses (pPLCγ2^+^, pSYK^+^ and either pBLNK^+^ or ppERK^+^) for all CLL and healthy samples demonstrated a clear separation of the healthy individuals' samples from the CLL patients' samples solely by virtue of the combined %pX^+^ values (**Fig. 2D**). Unlike other biomolecular factors utilized as diagnostic tools in CLL, the phosphorylation of BCR proximal signaling components unambiguously distinguishes aberrant signaling pathway behavior from healthy functionality.

### Multifactorial statistical modeling of CLL-specific BCR signaling behavior reveals key sources of dysfunction

The immunophenotypic diagnosis of CLL relies on the identification of CD5^+^CD3^−^CD23^+^CD20^low^ light chain-restricted lymphocytes in the blood or bone marrow of affected patients. Here, we use only phosphoresponses following BCR stimulation to reliably identify CLL patients. Partial-least square regression (PLSR) was applied to correlate the measured phosphoresponses with the disease status; creating a novel variable that accurately distinguishes and classifies pathological from healthy B cells.

**Figure 3:**
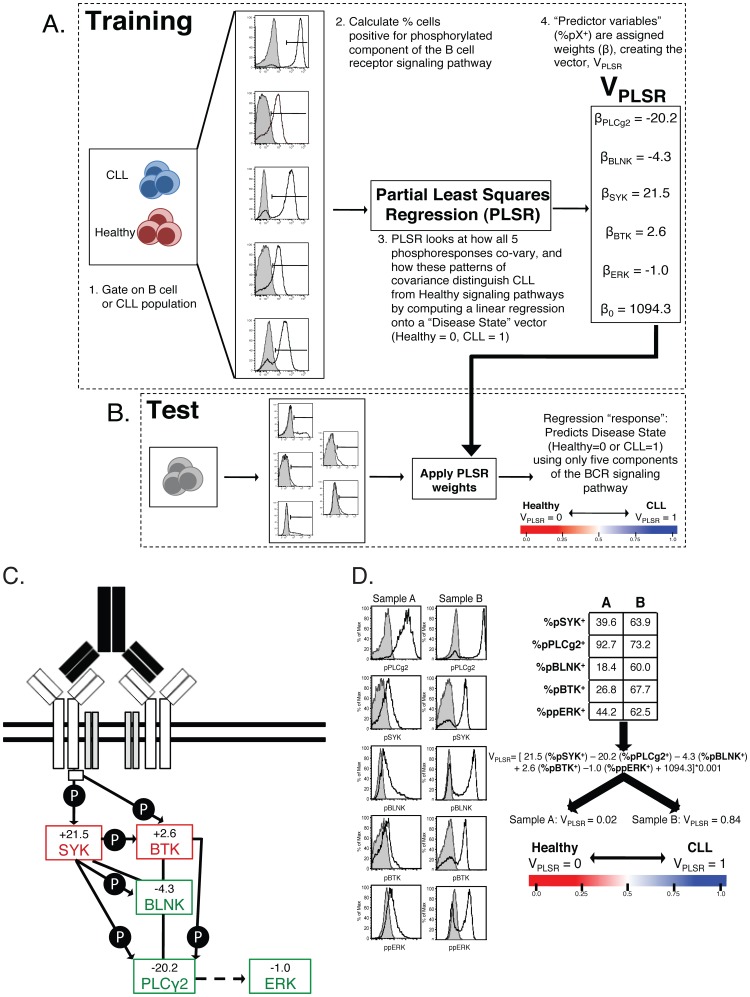
Statistical Analysis of CLL B-cell Phosphoprofile using Partial Least Squares Regression (PLSR) against disease status. This figure details our method for analyzing phosphoflow data by Partial Least Squares Regression (PLSR) against disease status: A Training: 1 CLL B cells or healthy B cells were isolated using the gating described in the methods section. 2 Phosphospecific antibodies allowed detection of the phosphoresponse for pPLCγ2, pSYK, pBTK, pBLNK and ppERK. The percentage of cells positive for each phosphospecific antibody was calculated based on the unstimulated histograms. These %pX^+^ provide the raw data for Partial Least Squares Regression analysis, PLSR. 3 PLSR was applied to best correlate a linear combination of these phosphoprofiles for each patient (all healthy controls and all CLL samples pooled together) with a variable defining disease status (arbitrarily set to 0 for healthy individuals and 1 for CLL patients). This step of the analysis is referred to as “Training” as the PLSR algorithm uses a subset of CLL B cells and Healthy B cells to model the covariance of each phosphoresponse. 4 The output of PLSR analysis is a linear combination V_PLSR_ with weights (β*_i_*) to each observed “predictor” variable (%pX*_i_* ^+^). The PLSR algorithm output attempts to find weights that best match the differences between healthy and CLL patient phosphoprofiles with disease status. B Test: Using the model (V_PLSR_) defined during the training phase, we tested the power of our model by its ability to correctly predict the disease status of independently-acquired CLL patients and healthy individuals. Phosphoresponses are measured and linearly-combined into a V_PLSR_ variable as specified by the training step. C BCR signaling diagram highlighting pathway-based understanding of the V_PLSR_ score and weights. D Example of V_PLSR_ predictive power: two samples (one CLL and one healthy control) were tested side-by-side. As predicted, V_PLSR_ helps discriminate their differences in phosphoresponses. As illustrated in Fig. 3A, %pX^+^ values over an unstimulated control are calculated and linearly combined to yield V_PLSR_ for the sample under consideration. Sample 1119, yields V_PLSR_ = 0.02, while Sample 1062, yields V_PLSR_ = 0.84, consistently with disease status (healthy and CLL, respectively).

**Figure 3A** depicts in detail the practical application of PLSR analysis of phosphoflow data. CLL B cells or healthy B cells were isolated using the gating strategy previously described. Phosphospecific antibodies allowed detection of bimodal phosphoresponses for pPLCγ2, pSYK, pBTK, pBLNK and ppERK. The percentage of cells positive for each phosphospecific antibody was calculated based on the unstimulated histograms. These %pX^+^ (X = pPLCγ2, pSYK, pBLNK, pBTK, ppERK) provide the raw data for PLSR analysis. PLSR correlates the magnitude of each patient's phosphoresponse (%pX^+^) with a variable defining disease status (0 for healthy individuals and 1 for CLL patients; 7 healthy and 67 CLL pooled together). The PLSR algorithm attempts to optimize weights (β, in **Fig. 3A, B**) to match the differences between healthy and CLL phosphoprofiles. The output of PLSR analysis is a linear combination, V_PLSR_, that applies weights to each observed “predictor” variable (%pX^+^). Thus, we created the following metric (V_PLSR_) that encodes Healthy vs. CLL BCR signaling behavior, given the inputs %pX^+^. (**Fig. 3C, D**):
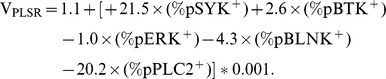
100% of the variance in phosphoresponse fluorescence intensity and 66% of the variance in clinical status is encompassed in this V_PLSR_ component. This weighted sum of individual phosphoresponses includes negative contributions from distal pPLCγ2, pBLNK and ppERK and positive contributions from more proximal kinases pSYK and pBTK. This implies that CLL B cells respond to BCR cross-linking with hypo-responsiveness for the distal pPLCγ2, pBLNK and ppERK, relatively to the proximal signaling events, compared to B cells from healthy individuals (**Fig. 3C**). By nature of the regression methodology, this dysregulation between proximal and distal signaling components is among the factors that most maximally distinguish a CLL BCR signaling pathway from that of a healthy B cell. PLSR on the most significant variable phosphoresponses (pPLCγ2 and pSYK) is in fact sufficient to deliver the same discrimination between CLL and healthy individuals (**Supp. Fig. S5**).

**Figure 4:**
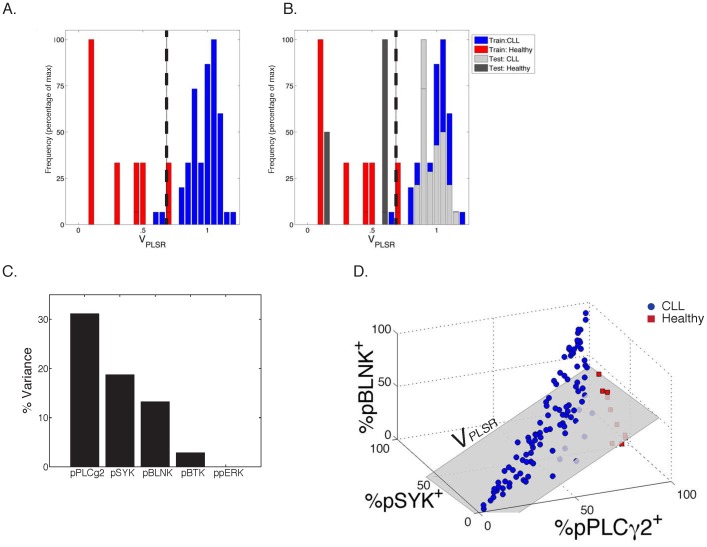
PLSR Model Results. A Distributions of V_PLSR_ scores for CLL and healthy samples from the training cohort. The x-axis shows the range of V_PLSR_ solutions, with healthy individuals significantly clustering on the lower extreme, and CLL patients' phosphoprofiles producing high V_PLSR_ scores (p<0.0001). This plot shows only patients included in the training set. The vertical black line at V_PLSR_ = 0.695 represents the optimized cutoff between CLL and healthy phosphoprofiles (see Supp. Fig. S7). B Distribution of V_PLSR_ scores for CLL and healthy samples from the training set (light and dark grey bars), overlaid to the V_PLSR_ distribution of the training set. The model is upheld as the test set data is correctly partitioned by disease status (p<0.0001). Root mean squared error (RMSE) measurements quantify the regression error, supporting the validity and applicability of the model to other datasets: RMSE_training_ = 0.1750; RMSE_test_ = 0.1576; RMSE_all_ = 0.1644. Training set: CLL = 67; Healthy = 7. Test set: CLL = 38; Healthy = 3. C Percent variance between the disease states, according to V_PLSR_ weight. Each phosphor-epitope individually accounts for the indicated percentage of the variability between CLL samples and healthy samples within the PLSR model. As shown, pPLCγ2^+^ has the strongest power in distinguishing healthy from CLL. D 3D-representation of V_PLSR_: a compendium of phosphoresponses best discriminating between CLL and healthy individuals. Each samples is represented as a datapoint, positioned in space according to the value of %pX^+^, for each axis. The grey plane corresponds to V_PLSR_ = 0.695, which best separates CLL from healthy individuals.

We then demonstrated how differential responsiveness in signaling responses discriminates between CLL and healthy individuals with high statistical significance (p<2×10^−5^). (**Fig. 4A**). Optimization of a V_PLSR_ threshold found that V_PLSR_ = 0.695 best discriminated between our training set of CLL and healthy individuals (**Supp. Fig. S6, S7**). Utilizing the V_PLSR_ model defined during our training phase, we tested the validity of our model using a “test set” from a separate CLL patient cohort (38 independently-acquired CLL patient samples, 3 healthy samples). We measured the stimulated signaling response of the test cohort, applied our V_PLSR_ classifier and found it 100% accurate (**Fig. 4B** **, Supp. Fig. S6B**). As pPLCγ2, pSYK and pBLNK account for 63% of the variability in the output, “response” variables (healthy vs. CLL) (**Fig. 4C**), we generated a 3D-representation of these phosphoresponses (**Fig. 4D**). This multidimensional visualization of the V_PLSR_ variable illustrates how CLL B cells respond with colinearity in these three critical phosphoresponses (pPLCγ2, pSYK and pBLNK) while healthy individuals display a deviation from this strict colinearity (grey plane in **Fig. 4D** **, Supp. Fig. S2A**).

Our multidimensional PLSR analysis demonstrates that the malignant properties of CLL B cells are evident and detectable in the B cell signaling response. PLSR analysis offered a robust filter to process multiple signaling readouts into a single discriminating variable, V_PLSR_. Comparison of V_PLSR_ with CD5 and CD20 MFI indicates independence of the BCR signaling dysregulation in CLL from these established phenotypic features of CLL B cells. (**Supp. Fig. S8**) This independence of V_PLSR_ from CD5 supports the ability of using the BCR signaling pathway phospho-signature as an independent measure of CLL disease status.

### Cell-to-cell heterogeneity in V_PLSR_ within the B cell population is both a characteristic hallmark of healthy samples and predictive of disease aggressiveness

**Figure 5:**
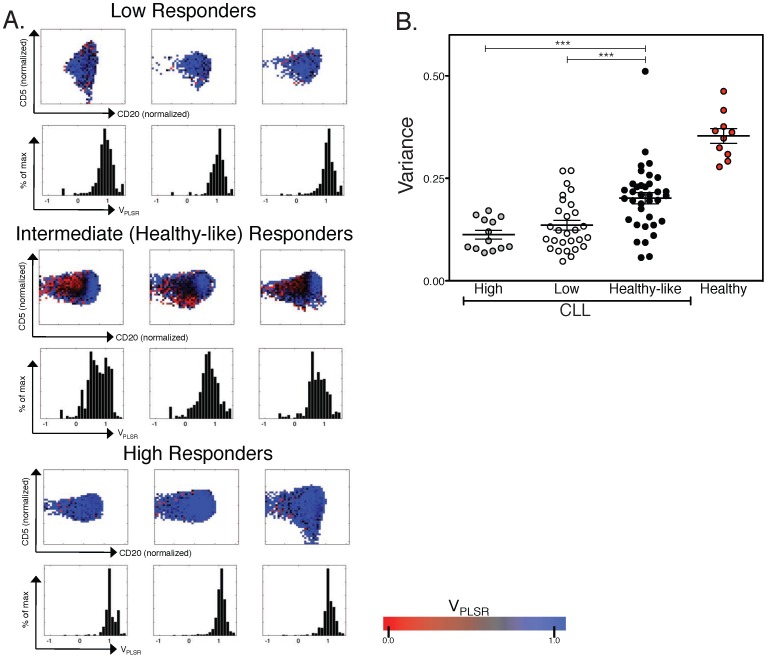
Cell-to-cell variability analysis for V_PLSR_ as a function of CD20 and CD5 abundance. A V_PLSR_ analysis of high responder, low responder, and healthy-like responder groups. Three representative patient samples are shown for each responder type. All CLL B-cell samples have a majority of their cellular population exhibiting a V_PLSR_>0.695 consistent with the CLL range (blue, as shown in the scale bar). Histograms represent the mean and spread of the V_PLSR_ values within each sample's B-cell population for low, high, and healthy-like responders. While low and high responders have a V_PLSR_ score centered around 1, the width of this peak is highly variable, but much more so in the healthy-like CLL. Healthy samples uniformly have a large variance of V_PLSR_ scores. B Evaluation of the heterogeneity within the samples as a measure of the variance of V_PLSR_ within the B cell population. Healthy-like responders exhibited a significantly larger variance compared to high or how responders (healthy-like vs. high: p = 0.0005; healthy-like vs. low: p = 0.0008; high vs. low: p = 0.23). The variance of V_PLSR_ within the B cell population of healthy samples is also shown.

Parsing of signaling responses in conjunction with CD5 and CD20 levels demonstrates heterogeneity of responsiveness within a CLL population. We applied cell-to-cell variability analysis (CCVA) [35], [36] to our single-cell phosphoprofiling measurements of CLL B cells. For each sample of stimulated B cells, CCVA parsed cells into subpopulations according to their abundance of CD20 and CD5 (**Fig. 5A**). Within each subpopulation, we computed the average V_PLSR_ and obtained the distribution V_PLSR_(CD20,CD5). We then estimated the variability of BCR signaling by computing the standard deviation *σ* of the distribution V_PLSR_(CD20,CD5): *σ (CD20, CD5)* quantifies the heterogeneity of BCR signaling conditioned by the abundance of CD20 and CD5. Using the definition of subgroups of patients based on their responsiveness as defined in **Figure 2A** (“low responders”, “intermediate, healthy-like responders” and “high responders”), we found that healthy-like responders were individuals whose B cells displayed significantly larger *σ (CD20, CD5) i.e.* larger variability in signaling compared to low- or high-responders (**Fig. 5B**).

## Discussion

The ability of CLL cells to defy apoptosis is the result of a series of signaling events that favor survival over cell death. Gene expression profiling analyses have demonstrated that normal B cells and CLL B cells differ by thousands of differentially expressed genes. However, only a relatively small number of genes can differentiate between CLL with mutated and unmutated IGHV genes [37], [38]. The CLL genes that distinguished between the two subtypes were enriched for genes that are modulated upon BCR stimulation, particularly in the unmutated samples. Additionally, the presence of stereotyped IGHV genes in a significant proportion of CLL patients further supports the hypothesis of chronic antigenic BCR stimulation [39]–[41]. A recent study revealed a mechanism of cell-autonomous BCR stimulation in CLL, totally independent of antigenic stimulation, but requiring the presence of distinct epitopes intrinsic to the BCR itself [19]. Such variation in the degrees of autonomous or antigen-dependent signaling response via the BCR might explain the variation in clinical behavior among CLL patients.

We postulated that a quantitative assay probing CLL-specific signaling signatures in a large patient cohort could distinguish between CLL and healthy B cells, and that specific signatures could correlate with the heterogeneity of the disease. Heterogeneity has been shown by phospho-flow cytometry in recently published work. A recent small-scale study of 11 CLL samples showed generally impaired CLL signaling responses to BCR stimulation compared to healthy B cells, and an association was noted between signaling ability and outcome [42]. In a separate study, phosphoprofiling of 23 CLL samples demonstrated that, while anti-IgM crosslinking alone produced minimal phosphoresponses, addition of H_2_O_2_ as a mean of signal amplification via tyrosine phosphatase inhibition could segregate patients' CLL cells in high and low responders, implying variability in the differential proximal BCR modulation within the clonal population. Furthermore, the apoptotic response to F-Ara-A exposure was directly correlated to the size of the H_2_O_2_-responsive population, indicating that there is a direct association between BCR signaling ability and apoptotic response [29].

Our methodology combining flow cytometry and multifactorial statistical analysis of phosphoresponse profiles for 105 CLL patients uncovered a robust signaling defect in CLL B cells. Though activation of B cells with anti-IgM and H_2_O_2_, a commonly used *in vitro* method of stimulation, likely does not perfectly reproduce *in vivo* conditions, it shifts the system into a state of maximal activation. This permits insights into signaling mechanisms that differentiate CLL from healthy B-cells. Multidimensional regression analysis (PLSR) of B cell phosphoresponses against disease status assigned positive weights for activation of proximal kinases (SYK and BTK) and negative weights for activation of distal kinases (PLCγ2, BLNK and ERK). The resulting PLSR variable from our study was found to strongly correlate with disease status (high for CLL versus low for healthy). Thus we analyzed the overall 5-dimensional phosphoprofile as a whole in order to uncover the defect in the pathologic CLL clone compared to healthy cells. Other groups have utilized similar methodologies to analyze multidimensional data, in which complex statistical models are summarized in a practical mathematical procedure [43], [44]. Yet, none have applied these approaches to flow cytometric data, nor focused on defined biochemical networks to uncover mechanistic properties.

The critical utility of the PLSR analysis lies in its ability to model and probe mechanistic differences between CLL and healthy B cell; it is not meant to be used as a diagnostic tool for CLL. Furthermore, the information contained in the PLSR model is independent from established prognosticators. In fact, our discovery may be of fundamental biological significance as PLCγ2 is a critical signaling regulator of B-cell activation, whose hypoactivation could be used as a biomarker for response to therapies targeting the BCR signaling pathway, in CLL and other lymphoproliferative disorders. Along those lines, Song et al. reported how total SYK and PLCγ2 phosphorylation upon dasatinib treatment (a SRC inhibitor) predicted the apoptotic response to the drug [45]. Previous studies of BCR signaling response may have failed to discover the *differential* pSYK vs pPLCγ2 defect that we report here, because it is not absolute. Indeed, we found that there exists vast variability in signaling behavior between patients, which prevents using PLCγ2 or SYK phosphorylation alone as a direct readout of BCR aberrant signaling. This is particularly significant for future studies as the identity of the dysfunctional regulators in CLL BCR signaling (possibly a differentially-regulated phosphatase) must carry more specificity towards pPLCγ2 inhibition compared to other kinases. Relating V_PLSR_ to other diagnostic and prognostic readouts, we found that those of worse outcome (treatment status, CD38 expression, cytogenetic defects associated with poor prognosis) did correlate, though with only modest statistical significance, with lower V_PLSR_ (data not shown).

Our study also confirmed bimodality in the phosphoresponse of CLL patients, as previously described by Palazzo et al. [29], here for each of the five kinases under study; this is characteristic of a globally-perturbed kinase network structure in CLL. Furthermore, while vast variability was observed between the stimulation signatures of different CLL patients, the tightly-controlled activation of the healthy donors was found to be robust, reproducible, and independent of healthy donor age (**Supp. Fig. S9**). This is particularly significant as access to blood samples from age-matched healthy donors can be limiting, yet unnecessary in the case of our methodology. Our study also identified two groups of patients, based on differential responsiveness within their CLL B cell population: patients whose majority of CLL cells strongly responded to antigenic activation were found to require treatment earlier and to harbor more frequently unmutated IGHV genes (both indicators of poor prognosis).

To conclude, this work demonstrates that a combination of phospho-specific flow cytometry and PLSR analysis can quantify how different neoplastic and benign cells behave within the same sample. Our multiparametric methodology can help identify mechanisms of dysregulation, that otherwise could never be explored by less sophisticated techniques. Hence, with minimal manipulation in a clinical setting, direct activation and probing of signaling responses with single–cell resolution by flow cytometry can yield new measurements to further classify subtypes of CLL with statistical significance.

## Supporting Information

Table S1 Patient Profiles and Clinical Characteristics.
(PDF)

Figure S1 Optimization and Validation of thaw, rest, and stimulation procedure. A. Rest time: Two CLL (blue) and two healthy donors (red) were rested for varied amount of time (as indicated on the x-axis), followed by a 4-min stimulation with anti-IgM and 3.3 mM hydrogen peroxide. These data demonstrate that B cells rapidly reach a steady-state of BCR-responsiveness after thawing and we conservatively chose a 2 hr-rest period for all our assays. B. H_2_O_2_ Titration: Four CLL patients (blue) and two healthy samples (red) were stimulated with anti-IgM and various concentrations of hydrogen peroxide (3.3 µM, 33 µM, 330 µM and 3.3 mM). %pBLNK_+_, %pPLC2_+_, %pSYK_+_ and %pBTK_+_ was used to measure the stimulation outcomes. We chose to use 3.3 mM H_2_O_2_ to boost the signaling responses of B cells in our assay. C. Stimulation conditions employed represent optimal setting for analysis of signaling mechanisms in CLL: Four experimental conditions and their effects on the signaling pathway response of CLL and healthy samples are shown. Data presented here justify the combined use of hydrogen peroxide and anti-IgM to achieve maximal discriminatory power among samples.
(TIFF)

Figure S2 Pairwise comparison of two-dimensional phosphoresponses. A %pX_+_ values for all CLL (blue) and healthy (red) samples. Each possible pairwise combination is shown. B MFI of cells responding with phosphorylation of X (X = PLC2, BLNK, SYK, BTK and ERK) in all possible pairwise combination. C Contour maps of the average B cell population: For each cohort, CLL and healthy, the cellular phosphoresponse fluorescence intensity values are averaged and viewed two dimensionally. Bimodality in the phosphoresponse of CLL B cells can be seen for 3 pairwise combinations (pBLNK vs pSYK, pPLCγ2 vs pBLNK and ppERK vs. pBTK). Healthy individuals show modest variability within a single population, while the CLL B-cells can be distinguished by their all-or-none response; a novel observation of BCR signaling pathway dynamics in CLL patients.
(TIFF)

Figure S3 Assessment of Apoptosis after 2-hour rest. A. Apoptosis measured with Annexin V and 7-AAD, herein double-positive cells used to identify the percentage of dead cells within the CD19+ B cells. There is no significant difference of the mean percent dead cells between high CLL responders, low CLL responders, or healthy PBMCs. B. No correlation exists between %pPLCg2 and the percentage of dead B cells (R_2_ = 0.07, p-value = 0.44, not significant).
(TIFF)

Figure S4 Correlating CLL patients' phosphoresponses with treatment status. Only PLC2 phosphoresponse is significantly different for treated and untreated patients (***: p<0.001).
(TIFF)

Figure S5 PLSR using only %pSYK_+_ and %pPLCγ2_+_ illustrates how these two factors, which accounted for the majority of the variance in the 5-phosphoresponse PLSR, are sufficient in partitioning CLL from healthy samples. A V_PLSR_(pPLCγ2, pSYK) equation. This new PLSR variable only takes into account a sample's %pPLCγ2+ and %pSYK+ values. Note the similarity in the PLSR weights between this equation and the original V_PLSR_. B BCR signaling diagram highlighting pathway-based understanding of the V_PLSR_ score and weights. C Plot of %pPLCγ2_+_ vs %pSYK_+_ for all samples. Datapoints represent individual samples, blue denotes CLL patients, red denotes healthy individuals. The dashed line represents the V_PLSR_(pPLCγ2, pSYK) variable solved such that the disease states are maximally differentiated. D Frequency distribution of V_PLSR_(pPLCγ2, pSYK) values for all CLL and healthy controls. This variable is able to distinguish samples by disease state (p<0.0001).
(TIFF)

Figure S6 Two-dimensional representation of CLL vs Healthy discrimination based on PLSR values. A. Training Set CLL and Healthy individuals. V_PLSR_ partitioning line (V_PLSR_ = 0.695) is shown in black. B. Training and Test set. V_PLSR_ discriminating line correctly partitions the test data (p<0.0001) by disease state.
(TIFF)

Figure S7 Cross Validation Justifies PLSR Power and the Use of Other Datasets. A Using Leave-One-Out Cross Validation, the RMSE remains small, and separation between the BCR signaling responses of CLL patients and healthy individuals remains strong. The results here are visualized using a cumulative distribution function plot, showing that the separation between the two disease states is consistent. B Using Leave-One-Out Cross Validation, we can determine frequency of error in the PLSR discrimination between disease states. A cutoff of V_PLSR_ scores to distinguish CLL vs. healthy is optimized based on these error frequencies: cutoff = 0.695. At this cutoff, <1/10 Healthy samples are falsely defined as CLL, and 4/105 CLL samples are falsely defined as Healthy based on their V_PLSR_ score. C Validation of PLSR Model. The relationship between the number of regression components included and the root mean squared error (RMSE) is shown here. Five components, or latent variables, are used to minimize error without overfitting.
(TIFF)

Figure S8 V_PLSR_ does not correlate with the abundance of CD5 (A) and CD20 (B) in B cells for CLL patients and healthy individuals.
(TIFF)

Figure S9 The ability of V_PLSR_ to discriminate between CLL and healthy patients is independent of donor age. Our original healthy donors (used for training and testing the V_PLSR_) were not agematched (HD median age: 36 year-old, CLL median age: 66 year-old). To test if the donor age affected the reliability of our method, we collected a separate cohort of agematched healthy donors (median age = 69 year-old) and young healthy donors (median age = 24 year-old). We applied the BCR stimulation and V_PLSR_ processing protocol (as outlined in our methods) to (n = 7) CLL patients, (n = 7) age-matched healthy donors and (n = 6) young healthy donors. A) V_PLSR_ of CLL patients (blue) and healthy donors (red) from “training” and “test” cohorts as a function of age. Note the lack of correlation (CLL: R_2_ = 0.004; Healthy: R_2_ = 0.03, n.s.), which justifies using samples collected from non age-matched healthy donors. B) Stimulation and analysis protocols (Fig. 3) were applied to a novel group of CLL patients (blue), age-matched healthy donors (dark red), and younger healthy donors (red). Independent of age, our methodology discriminates between disease states in this new experimental cohort. This figure is representative of two repeats.
(TIFF)
