# Supplementary material for: Multidimensional Single-Cell Analysis of BCR Signaling Reveals Proximal Activation Defect As a Hallmark of Chronic Lymphocytic Leukemia B Cells
Source: PLoS One. 2014 Jan 29;9(1):e79987. doi: 10.1371/journal.pone.0079987 (PMC3906024; doi:10.1371/journal.pone.0079987)
Supplement: Table S1 — Patient Profiles and Clinical Characteristics. (PDF) [file pone.0079987.s001.pdf]

## Table 1.

| Code | % Zap70+ cells | % CD38+ cells | Cytogenetics | V genes | TTFT (months) |
|---|---|---|---|---|---|
| **1001** | 2.00 | 0.50 | del 13q | mutated | 85.9 |
| **1002** | 50.00 | 1.20 | del 13q | unmutated | 1.2 |
| **1003** | 8.00 | 0.90 | del 13q | mutated | 53.6 |
| **1004** | 80.00 | 60.40 | del 13q | unmutated | 34 |
| **1005** | 1.00 | 7.20 | del 13q, trisomy 12 | mutated | Not reached |
| **1006** | 75.00 | 16.10 | trisomy 12 | unmutated | Not reached |
| **1007** | 2.00 | 1.00 | del 13q | mutated | 54.1 |
| **1008** | 82.00 | 55.10 | trisomy 12 | unmutated | 0 |
| **1009** | 66.00 | 23.20 | normal | unmutated | 0.2 |
| **1010** | 76.00 | 97.80 | trisomy 12 | unmutated | 12.8 |
| **1012** | 1.00 | 0.50 | del 13q | mutated | 125 |
| **1013** | 6.00 | 28.70 | trisomy 12 | unmutated | 31 |
| **1014** | 53.00 | 3.70 | del 17p | unmutated | 30.4 |
| **1015** | 6.00 | 4.00 | normal | mutated | Not reached |
| **1016** | 2.00 | 34.70 | normal | mutated | Not reached |
| **1017** | 30.00 | 24.60 | normal | mutated | Not reached |
| **1018** | 53.00 | 24.30 | del 11q, del 13q, trisomy 12 | unmutated | 12.4 |
| **1019** | 11.00 | 74.10 | trisomy 12 | unmutated | Not reached |
| **1021** | 16.00 | 2.60 | del 13q | unmutated | 13.2 |
| **1022** | 87.00 | 76.40 | del 13q | unmutated | 38.7 |
| **1023** | 64.00 | 39.50 | del 13q, del 11q | mutated | 4.1 |
| **1024** | 58.00 | 16.80 | del 13q | unmutated | 40.9 |
| **1025** | 71.00 | 51.50 | trisomy 12 | unmutated | 45 |
| **1026** | 11.00 | 2.80 | n/a | unmutated | 9.6 |
| **1027** | 59.00 | 35.80 | del 13q | unmutated | 0 |
| **1028** | 15.00 | 3.90 | del 11q, del 13q | unmutated | 20 |
| **1029** | 41.00 | 68.90 | del 11q, del 13q | mutated | 55 |
| **1030** | 32.00 | 19.00 | trisomy 12 | unmutated | Not reached |
| **1031** | 3.00 | 2.20 | del 13q | mutated | 34 |
| **1032** | 4.00 | 2.20 | normal | n/a | Not reached |
| **1033** | 35.00 | 2.50 | del 11q, del 13q | unmutated | 20.9 |
| **1034** | 50.00 | 90.70 | del 13q | mutated | 16.1 |
| **1035** | 38.00 | 2.20 | del 13q | unmutated | 45 |
| **1036** | 1.00 | 3.30 | del 13q | n/a | Not reached |
| **1037** | 34.00 | 2.80 | del 13q, del 17p | mutated | Not reached |
| **1038** | 2.00 | 1.90 | del 13q | mutated | Not reached |
| **1039** | 19.00 | 1.40 | del 17p | unmutated | 29.1 |
| **1040** | 16.00 | 33.00 | n/a | unmutated | 66.2 |
| **1041** | 46.00 | 19.70 | normal | unmutated | Not reached |
| **1042** | 14.60 | 73.70 | del 11q, del 13q | unmutated | 71.1 |
| **1043** | 45.80 | 31.50 | del 11q | unmutated | Not reached |
| **1044** | 17.10 | 3.90 | normal | mutated | Not reached |
| **1045** | 26.00 | 1.20 | del 13q | unmutated | Not reached |
| **1046** | 30.50 | 44.50 | del 17p | unmutated | 3 |

| | | | | | |
|---|---|---|---|---|---|
| **1047** | 33.80 | 2.54 | n/a | mutated | 26.9 |
| **1048** | 27.60 | 0.59 | del 13q | unmutated | Not reached |
| **1049** | 20.80 | 2.08 | del 13q | mutated | Not reached |
| **1050** | 17.80 | 1.00 | normal | mutated | Not reached |
| **1051** | 22.90 | 0.78 | del 13q | mutated | Not reached |
| **1052** | 16.60 | 0.70 | del 13q14 | mutated | Not reached |
| **1053** | 25.80 | 1.75 | normal | mutated | Not reached |
| **1054** | 22.40 | 0.87 | normal | unmutated | 70.8 |
| **1055** | 17.60 | 0.80 | normal | unmutated | 55.5 |
| **1056** | 20.70 | 53.90 | del 13q14, loss of p53 | unmutated | 26.3 |
| **1057** | 14.40 | 20.30 | del 13q14 | mutated | 6.3 |
| **1058** | 24.30 | 3.82 | n/a | mutated | Not reached |
| **1059** | 26.50 | 1.50 | del 11q | unmutated | 69.9 |
| **1061** | 8.58 | 1.19 | n/a | mutated | Not reached |
| **1062** | 18.20 | 2.62 | del 17p | unmutated | 7.4 |
| **1063** | 29.30 | 1.13 | n/a | mutated | Not reached |
| **1064** | 23.00 | 1.27 | normal | mutated | Not reached |
| **1065** | 21.10 | 0.33 | del 13q | mutated | 196.3 |
| **1066** | 15.30 | 76.40 | normal | unmutated | Not reached |
| **1067** | 33.60 | 5.28 | n/a | unmutated | Not reached |
| **1068** | 15.70 | 2.70 | del 13q | mutated | 233.8 |
| **1069** | 25.80 | 0.47 | del 13q | mutated | Not reached |
| **1070** | 22.70 | 21.10 | normal | unmutated | 78.7 |
| **1071** | 36.10 | 1.15 | del 13q | mutated | 191.8 |
| **1072** | 12.50 | 2.32 | normal | mutated | Not reached |
| **1073** | 35.80 | 4.83 | del 13q | unmutated | 25.5 |
| **1074** | 38.20 | 6.04 | del 17p | mutated | 196 |
| **1075** | 6.30 | 6.97 | del 17p, del 11q, monosomy 6, loss of p53 | unmutated | 15.7 |
| **1076** | 27.10 | 1.40 | del 13q, del 6q, del 11q and loss of p53 | mutated | Not reached |
| **1077** | 19.50 | 5.10 | del 13q and loss of p53 | mutated | Not reached |
| **1078** | 17.10 | 35.70 | trisomy 12, 1p38 rearrangement | unmutated | 7.3 |
| **1079** | 31.30 | 39.50 | del 13q | unmutated | 16.5 |
| **1080** | 15.00 | 1.62 | del 13q | mutated | 6.4 |
| **1081** | 39.80 | 61.00 | del 13q, trisomy 18 | mutated | 32.5 |
| **1082** | 19.30 | 5.79 | trisomy 12 | mutated | 10.7 |
| **1083** | 45.50 | 89.20 | trisomy 12 | unmutated | 63.5 |
| **1084** | 4.60 | 67.80 | trisomy 12 | mutated | Not reached |
| **1085** | 16.60 | 0.26 | normal | mutated | Not reached |
| **1086** | 35.00 | 0.08 | n/a | mutated | Not reached |
| **1087** | 35.60 | 0.15 | del 13q | mutated | Not reached |
| **1088** | 35.50 | 0.83 | normal | mutated | Not reached |
| **1089** | 40.20 | 53.40 | normal | unmutated | Not reached |
| **1090** | 12.70 | 1.40 | del 13q | mutated | Not reached |
| **1091** | 19.80 | 0.30 | del 13q and loss of p53 | mutated | 36.3 |
| **1092** | 15.90 | 16.10 | trisomy 12 | mutated | Not reached |

!

| | | | | | |
|---|---|---|---|---|---|
| **1093** | 29.20 | 7.53 | del 13q | mutated | 36.3 |
| **1095** | 26.40 | 53.00 | del 13q and del 11q | mutated | 27.5 |
| **1096** | 7.50 | 8.97 | complex cytogenetics including loss of p53 | unmutated | Not reached |
| **1097** | 45.70 | 72.60 | n/a | mutated | 57.4 |
| **1098** | 18.40 | 50.60 | del 13q | unmutated | 60.6 |
| **1100** | n/a | n/a | trisomy 12 | unmutated | Not reached |
| **1101** | 13.20 | 1.46 | normal | mutated | Not reached |
| **1102** | 18.70 | 0.39 | del 13q | mutated | Not reached |
| **1103** | 32.60 | 0.43 | del 13q | mutated | Not reached |
| **1104** | 7.08 | 0.10 | del 13q | mutated | Not reached |
| **1105** | 1.39 | 0.12 | n/a | mutated | Not reached |
| **1106** | 3.88 | 0.08 | normal | n/a | Not reached |
| **1107** | 11.80 | 0.23 | trisomy 12 | unmutated | Not reached |
| **1108** | 8.46 | 0.26 | del 13q and loss of p53 | n/a | Not reached |
| **1109** | 1.91 | 0.04 | del 13q | mutated | Not reached |
| **1110** | n/a | n/a | normal | unmutated | Not reached |
