# Supplementary figures and images for: Multidimensional Single-Cell Analysis of BCR Signaling Reveals Proximal Activation Defect As a Hallmark of Chronic Lymphocytic Leukemia B Cells
Source: PLoS One. 2014 Jan 29;9(1):e79987. doi: 10.1371/journal.pone.0079987 (PMC3906024; doi:10.1371/journal.pone.0079987)

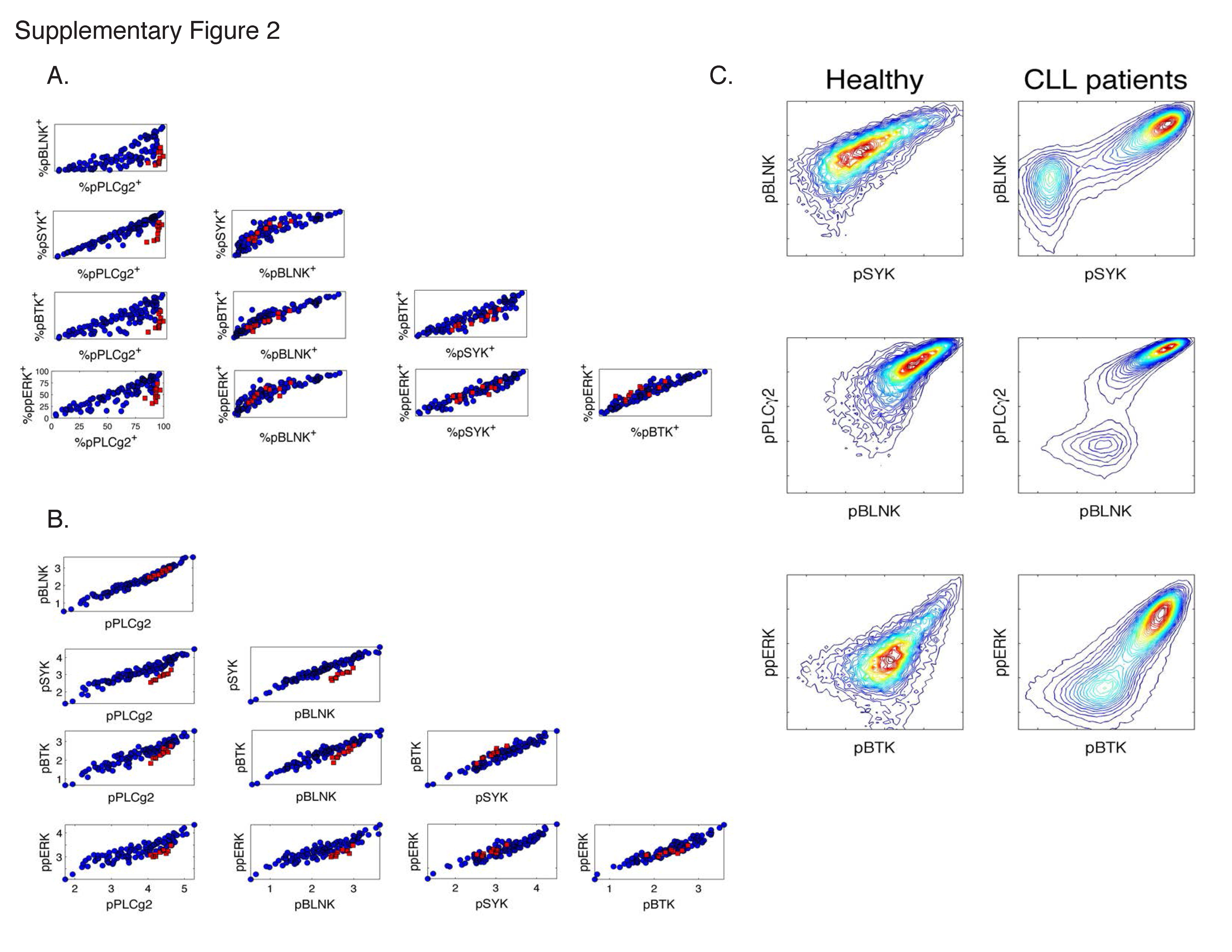

Supplement: Figure S2 — Pairwise comparison of two-dimensional phosphoresponses. A %pX+ values for all CLL (blue) and healthy (red) samples. Each possible pairwise combination is shown. B MFI of cells responding with phosphorylation of X (X = PLC2, BLNK, SYK, BTK and ERK) in all possible pairwise combination. C Contour maps of the average B cell population: For each cohort, CLL and healthy, the cellular phosphoresponse fluorescence intensity values are averaged and viewed two dimensionally. Bimodality in the phosphoresponse of CLL B cells can be seen for 3 pairwise combinations (pBLNK vs pSYK, pPLCγ2 vs pBLNK and ppERK vs. pBTK). Healthy individuals show modest variability within a single population, while the CLL B-cells can be distinguished by their all-or-none response; a novel observation of BCR signaling pathway dynamics in CLL patients. (TIFF) [file pone.0079987.s003.tiff]

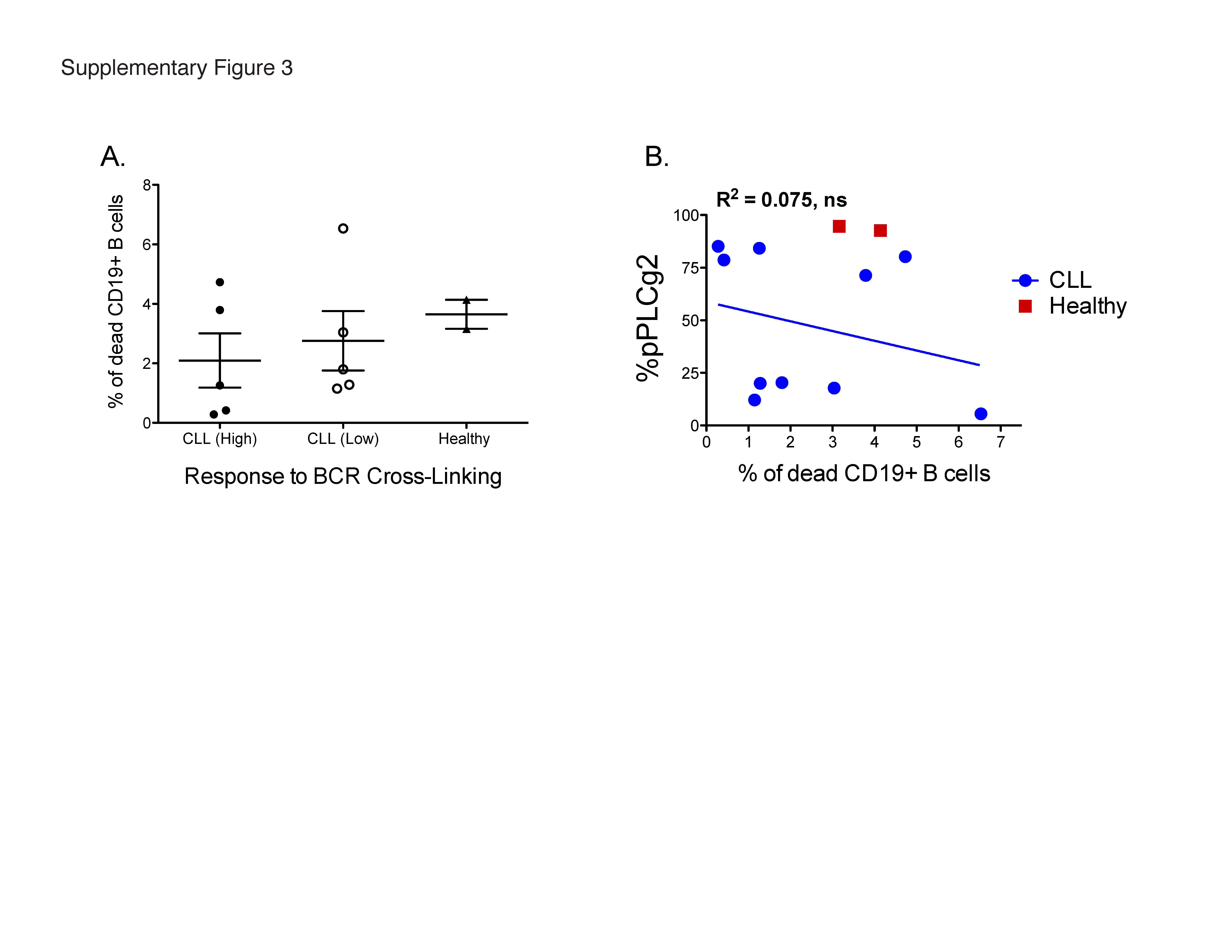

Supplement: Figure S3 — Assessment of Apoptosis after 2-hour rest. A. Apoptosis measured with Annexin V and 7-AAD, herein double-positive cells used to identify the percentage of dead cells within the CD19+ B cells. There is no significant difference of the mean percent dead cells between high CLL responders, low CLL responders, or healthy PBMCs. B. No correlation exists between %pPLCg2 and the percentage of dead B cells (R2 = 0.07, p-value = 0.44, not significant). (TIFF) [file pone.0079987.s004.tiff]

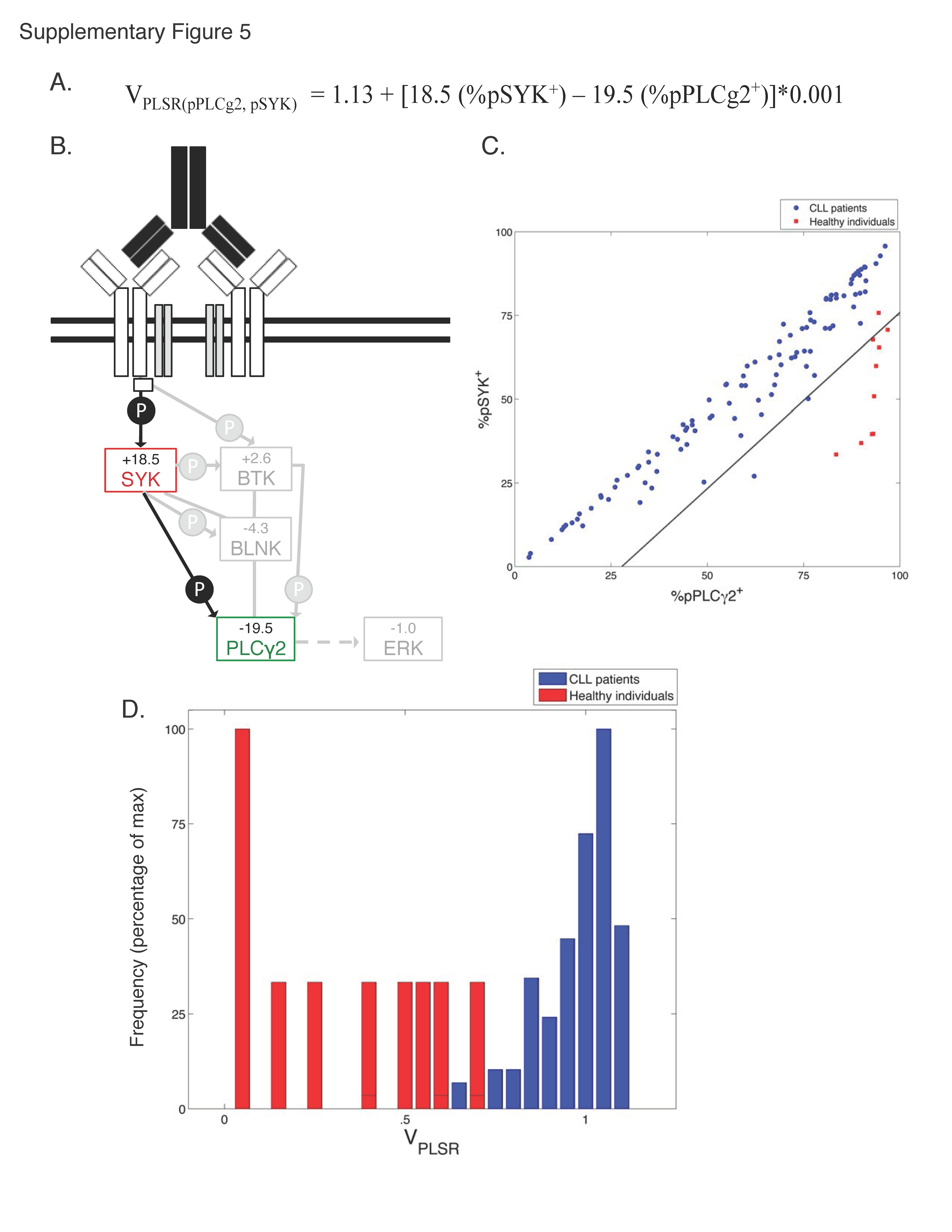

Supplement: Figure S5 — PLSR using only %pSYK+ and %pPLCγ2+ illustrates how these two factors, which accounted for the majority of the variance in the 5-phosphoresponse PLSR, are sufficient in partitioning CLL from healthy samples. A VPLSR(pPLCγ2, pSYK) equation. This new PLSR variable only takes into account a sample's %pPLCγ2+ and %pSYK+ values. Note the similarity in the PLSR weights between this equation and the original VPLSR. B BCR signaling diagram highlighting pathway-based understanding of the VPLSR score and weights. C Plot of %pPLCγ2+ vs %pSYK+ for all samples. Datapoints represent individual samples, blue denotes CLL patients, red denotes healthy individuals. The dashed line represents the VPLSR(pPLCγ2, pSYK) variable solved such that the disease states are maximally differentiated. D Frequency distribution of VPLSR(pPLCγ2, pSYK) values for all CLL and healthy controls. This variable is able to distinguish samples by disease state (p<0.0001). (TIFF) [file pone.0079987.s006.tiff]

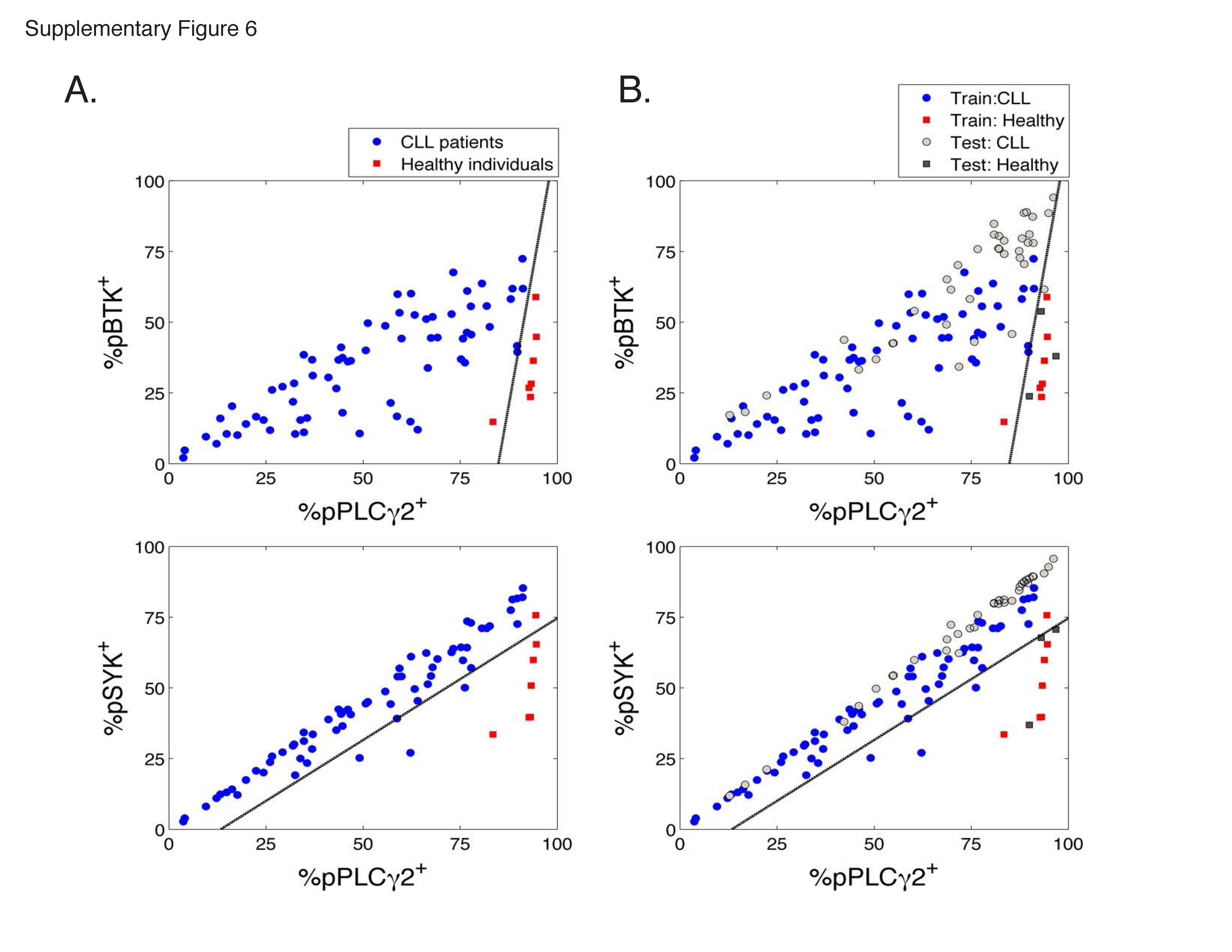

Supplement: Figure S6 — Two-dimensional representation of CLL vs Healthy discrimination based on PLSR values. A. Training Set CLL and Healthy individuals. VPLSR partitioning line (VPLSR = 0.695) is shown in black. B. Training and Test set. VPLSR discriminating line correctly partitions the test data (p<0.0001) by disease state. (TIFF) [file pone.0079987.s007.tiff]
